# Modern Sociology

**Published:** 1899-07-22

**Authors:** 


### Modern Sociology.

### BOARDING-SHIPS AS BOARD SCHOOLS.

By a Correspondent.

Our national chiefs are at present, many of them, trying to impress on all classes the need of recruits for the navy. And not only so, but we may also say that all classes have responded spontaneously, and have been holding meetings to consider this problem under various aspects. We would offer a few suggestions, not indeed as a solution of this problem, but as pointing out some of its remote bearings towards the masses in the Midland Counties.

It may seem at first sight unnecessary to insist upon the fact that the youth of our nation are its jewels. But we have been so beset with jokes on the "age of obedient parents," that we are apt to fall short of the true meaning of the phrase. For surely with us, as with the Platonic Greeks, the two first duties of a citizen are to protect and to fight—or, as we put it, to "hold our own"—and it is pre-eminently our wives and children whom we swear "to have and to hold." Our boys, at least, never allow us to forget them, they give us no rest, we are ceaselessly legislating for them, both in school and out; the problem of their true welfare is ever before us. They are the sap of our nation. The Board of Trade with its six departments, viz., commerce, railways, marine, harbour, fisheries, and finance—our army and navy, our whole destiny, depends on our young people. Their well-being—physical, moral, and mental—ought to concern us deeply. Those who are not parents are, at least, brothers, uncles, or cousins, and we ought to strengthen the old English family feeling.

We need to bring the fact of the sea home to the Midlands—we Inlanders and the coast villagers are one people. A great deal has been done by the railway companies of late. Seaside holidays and "week-ends" have become matters of course. At the same time, there is a tendency all over England to unwise precipitation of inhabitants on some special spot. It is far from our intention to advocate anything like interference with parental responsibility, yet we think it would be good for the future of the commonwealth if centres of population, such as Derby, could be relieved of those of its members who are not yet needful to the efficiency of the district. We are always hearing of the cost of new Board schools, and in great industrial and manufacturing centres the problem where to put these schools is becoming more and more pressing. Just as the question of workmen's houses was at the bottom of the great engineering strike, so the question of school quarters depends on the house question.

The good effects of sea air on the development of the young has long been admitted. Thousands of pounds sterling are annually voted by public and private charities to procure this blessing for those who cannot afford it unassisted. The rich have long set the fashion of seaside boarding schools, and it is precisely this fashion which we would fain see extended to our artisans and mechanics. For this purpose land on the coast could be obtained at a price comparatively small. The usual school pence could be charged to parents, and, in addition, the sum which the child would cost its family if at home would be paid to the school bursary. Possibly the Passmore Edwards Home just opened at Herne Bay may furnish some data as to the relative needful expenses to be incurred. These schools could be started by private companies in the first instance, and then taken over by Government. The saving in price as to site would go towards the primary cost of starting them. Possibly they might be built on Crown lands, at a nominal rent. It would be necessary, of course, to arrange with the great railway companies for free passes for parents and children. The opening up of new centres of resort on our coasts would offer a substantial inducement, and the relief from overcrowding at Margate, Ramsgate, Blackpool, &c., on Bank Holidays would be only one of the many incidental blessings of this scheme. So far we have thought merely of the indirect, though obvious results in improved physique, mental and moral tone, which could not fail to follow in the train of a movement such as this. No more alleys, back courts, smuts and dirt for the children of the slums means normal development of the senses as well as of the muscular system. This would minimise the need for oculists, also for surgical appliances. There would be fewer hospitals to keep up, and convalescent homes would be needless.

But alongside of these general advantages to the community at large liking for the sea and the seafaring life and industries must duly follow. Little brothers and sisters at home would long to grow up to have their turn as boarders in the Coast-Schools. Rowing, swimming, diving, trawling, &c., would divide the affection of boys with cricket and football. Boat-building clubs would soon be the rage. Carpentering, turning, netting, and other crafts would receive an impetus natural enough, and the need for their artificial culture would be lessened, with its enormous attendant expenses and temptations to patronage. But, pending the suggested change, would it not be possible to utilise old vessels by floating them and rigging them out to receive contingents of school boys after the manner of training ships? Such vessels would save all expense of rent, site, and building. Boys already in favour of a sailor's career could be chosen to form the first experimental contingents. The sea coast permanent-schools could be built gradually as occasion demands, and the boarding-vessels could be resumed by the Board of Trade or handed over direct to the Navy for training purposes.

Thus we should have a perpetual circulation of material from the Midlands to the Coasts, returning with renewed vitality to energise the forces of the country, or to man our traders and battle ships. This circulation of energy is to the nation what the circulation of the blood is to the individual. Let us subdue nature by obeying her laws.

